# Pathological Mechanisms Involved in Epidermolysis Bullosa Simplex: Current Knowledge and Therapeutic Perspectives

**DOI:** 10.3390/ijms25179495

**Published:** 2024-08-31

**Authors:** Mbarka Bchetnia, Julie Powell, Catherine McCuaig, Anne-Marie Boucher-Lafleur, Charles Morin, Audrey Dupéré, Catherine Laprise

**Affiliations:** 1 Département des Sciences Fondamentales, Université du Québec à Chicoutimi, Saguenay, QC G7H 2B1, Canada; mbarka.bchetnia@crchudequebec.ulaval.ca (M.B.); amblafle@uqac.ca (A.-M.B.-L.); 2 Centre Intersectoriel en Santé Durable, Saguenay, QC G7H 2B1, Canada; 3 CHU Sainte-Justine, Montreal, QC H3T 1C5, Canada; julie_powell.med@ssss.gouv.qc.ca (J.P.); med@ssss.gouv.qc.ca (C.M.); 4 Centre Intégré Universitaire de Santé et de Services Sociaux du Saguenay-Lac-Saint-Jean, Hôpital Universitaire de Chicoutimi, Saguenay, QC G7H 7K9, Canada; chamorinmd87@icloud.com (C.M.); audreydupere@videotron.ca (A.D.)

**Keywords:** epidermolysis bullosa simplex, expression profile, *KRT5*, *KRT14*, inflammation

## Abstract

Epidermolysis bullosa (EB) is a clinically and genetically heterogeneous group of mechanobullous diseases characterized by non-scarring blisters and erosions on the skin and mucous membranes upon mechanical trauma. The simplex form (EBS) is characterized by recurrent blister formation within the basal layer of the epidermis. It most often results from dominant mutations in the genes coding for keratin (K) 5 or 14 proteins (*KRT5* and *KRT14*). A disruptive mutation in *KRT5* or *KRT14* will not only structurally impair the cytoskeleton, but it will also activate a cascade of biochemical mechanisms contributing to EBS. Skin lesions are painful and disfiguring and have a significant impact on life quality. Several gene expression studies were accomplished on mouse model and human keratinocytes to define the gene expression signature of EBS. Several key genes associated with EBS were identified as specific immunological mediators, keratins, and cell junction components. These data deepened the understanding of the EBS pathophysiology and revealed important functional biological processes, particularly inflammation. This review emphasizes the three EBS subtypes caused by dominant mutations on either *KRT5* or *KRT14* (localized, intermediate, and severe). It aims to summarize current knowledge about the EBS expression profiling pattern and predicted molecular mechanisms involved and to outline progress in therapy.

## 1. Introduction

**Figure 1:**
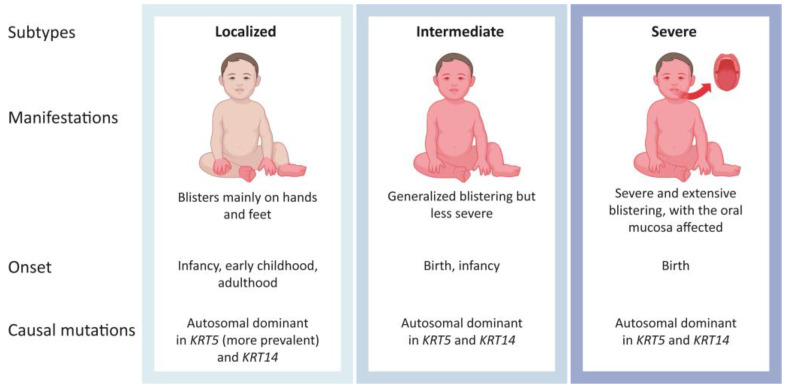
Epidermolysis bullosa simplex (EBS) main subtypes.

Epidermolysis bullosa simplex (EBS) affects the skin, a vital organ with a dynamic and complex organization protecting the body from stress conditions [1,2]. Most cases of EBS result from autosomal dominant mutations affecting either keratin 5 (*KRT5*) or *KRT14* [3]. Under normal conditions, the corresponding proteins, keratin 5 (K5) and 14 (K14), form heterodimers assembling together into intermediate filaments (IFs) [3]. In EBS, causal mutations can impact the structure of these proteins, causing keratin aggregates in keratinocytes, triggering endoplasmic reticulum stress and leading to the release of pro-inflammatory molecules [4]. Intraepidermal ruptures occur and lead to loss of tissue integrity and severe blistering of the epidermis upon mechanical trauma or traction [5,6,7,8]. A schematic representation of the structural impairments leading to EBS symptoms can be visualized by Li et al. [9]. The keratin defects in EBS also trigger itching, causing further discomfort while also yielding new lesions by scratching the skin and, thus, promoting inflammation [10,11]. The severity of EBS is highly variable with three main clinical subtypes: localized EBS (EBS-loc), intermediate EBS (EBS-intermed), and severe EBS (EBS-sev) [12,13]. Figure 1 represents the characteristics of these different subtypes. Rare subtypes of EBS exist, for which other causal mutations were identified in several genes (plectin (*PLEC*), Kelch-like member 24 (*KLHL24*), dystonin (*DST*), exophilin-5 (*EXPH5*), and CD151 molecule (*CD151*)) [11,12]. 

To deepen the characterization of the molecular mechanisms underlying the EBS phenotype, several gene expression profiling studies were performed and reported varying and sometimes conflictual findings [14,15,16,17,18,19,20,21,22,23,24], highlighting the need to discuss these results in a single paper. Expression profiles are tissue-specific, thus allowing an accurate representation of pathway dysregulation in the infected tissue of the EBS patients—the skin [25]. Inflammation mediators and other components critical for immune response and skin barrier homeostasis, including keratinocyte proliferation, differentiation, and migration, are highly upregulated in many expression profile studies [14]. The common agreement that surfaced is the potential implication of inflammation in EBS pathogenesis [19,26,27] as well as the possibilities of efficient treatment development.

This review gathers and discusses the findings of EBS transcriptomic studies regarding three main clinical subtypes (EBS-loc, EBS-intermed, EBS-sev). Expression studies performed under normal conditions were selected, with special attention to those also proposing therapeutic avenues. The following keywords were used to search the Pubmed database: epidermolysis bullosa, gene expression, transcriptomic analysis, gene therapy, and EBS treatment. 

## 2. EBS Gene Expression Profile Studies

### 2.1. Increase of Inflammation Components

Inflammatory processes are triggered by an immune system disturbance generally caused by tissue injury, infection, or other stresses. A variety of immune cell types migrate, proliferate, and produce cytokines, interleukins (ILs), and interferons (IFNs) in an orchestrated manner at the inflammation site [28,29]. Aside from the keratin defaults triggering EBS, it becomes clear that this disorder is associated with inflammation as reported by transcriptomic analysis. Various signaling inflammatory pathways were shown to be involved in the EBS pathomechanisms.

#### 2.1.1. c-Jun N-Terminal Kinase (JNK) Stress Pathway

In the skin, keratinocytes produce pro-inflammatory cytokines IL-1α and IL-1β and their receptors [30]. These ILs activate keratinocytes in various pathological conditions and upon tissue damage [14]. IL-1β precursor is present in the cytoplasm and, after injury, is released and activates signal transduction pathways in surrounding cells [4]. IL-6 stimulates keratinocyte growth and migration in vitro mediate local inflammation and modulate immune response upon wounding along with IL-1β [31]. 

Transcriptome analysis revealed that IL-1β and IL-6 mRNA levels were upregulated in K5−/− mouse skin [14]. Increased expression of IL-1β was also shown in keratinocytes derived from one patient with EBS-sev [23,30]. Upregulation of IL-6, IL-8, and IL-10 was also observed in the epidermis of patients with either *KRT5* or *KRT14* mutations [24]. This is consistent with local inflammation that can accompany EBS lesions. The c-Jun N-terminal kinase (JNK) stress pathway is activated through IL-1β in an autocrine manner [30]. This activates the transcription of AP-1 responsive genes such as matrix metalloproteinase 1 (*MMP1*), kallikrein 7 (*KLK7*), rho guanine nucleotide exchange factors (*ARHGEF* family), and *KRT14* and *IL-1β* themselves by positive feedback [20]. When EBS is caused by *KRT14* mutations, expression of the dominantly interfering mutated allele would increase through this mechanism and potentially aggravate the phenotype. Increased expression of MMPs (1, 7, 9, 13, 19) in EBS-sev keratinocytes was reported, as well as KLKs (5–8, 10–11, 13–14), indicating that various genes encoding matrix-degrading proteins are targets of the IL-1β pathway [21]. KLK5 and KLK7 differential expression was validated on the protein level by Western blot [21]. This highlights the role of inflammation in exacerbating the EBS phenotype, particularly in EBS-sev.

#### 2.1.2. IFN-γ Inflammatory Signaling Pathway

IFNs are major mediators of inflammation [32]. They are pleiotropic cytokines with antiviral, antitumor, and immunomodulatory functions and act as major effectors of the immune response [33]. Patients with EBS display higher systemic levels of inflammatory mediators such as IFN-γ, primarily secreted by NK cells and activated T lymphocytes [4]. Badowski et al. showed that the addition of IFN-γ to EBS-sev keratinocytes promotes the formation of protein aggregates, decreases cell–cell junctions, delays wound closure, and reduces cell proliferation as part of an immune antiviral response [34]. IFN-γ increases the phospho-Tyr701 signal transducer and activator of transcription (STAT) 1, which lies downstream in the IFN-γ inflammatory signaling pathway [35], as well as K17 as a stress response to reduce the dominant negative effect of mutated K14 possibly by copolymerizing with K5 in vivo [21,36].

#### 2.1.3. Phosphatidylinositol 3-Kinase (PI3K)-Protein Kinase B (Akt)-mTOR Pathway

The mechanistic target of rapamycin (mTOR) is a member of the phosphatidylinositol 3-kinase (PI3K)-related kinase family. It is stimulated by the protein kinase B (Akt), immediately downstream of PI3K. The PI3K/Akt/mTOR pathway regulates cell growth, proliferation, motility, and survival [37]. A recent study carried out transcriptomic analysis in EBS patients to characterize the molecular pathways activated in the EBS-blistered epidermis. Their findings revealed that IL-6, IL-8, and IL-10 were upregulated in the epidermis of EBS patients, and predicted upstream regulators included TNF-a, IL-1b, IL-2, IL-6, PI3K, and mTOR. They hypothesized that this signaling process is one of the major activated pathways in EBS [24].

#### 2.1.4. Wnt-Receptor Signaling Pathway

Enrichment clustering of dysregulated genes in whole-transcriptome microarray analysis of an EBS-sev cell line highlighted the upregulation at the RNA and protein levels of *WNT5a*, encoding for one of the so-called noncanonical Wnt ligands [22]. During normal development, WNT5a is secreted and directs the migration of target cells along concentration gradients. Dysregulation of WNT5a signaling facilitates invasion by multiple tumor types into contiguous tissues [38]. It is known that patients with EBS-sev have a slightly higher risk of developing basal cell carcinoma (BCC) [4], and dysregulation in WNT5a signaling could contribute to this outcome. The roles of WNT5a in this context have not been fully investigated yet nor its impact on keratinocytes migration.

#### 2.1.5. Bone Morphogenetic Proteins Signaling (BMPs)

Bone morphogenetic proteins (BMPs) are signaling polypeptides of the transforming growth factor-β (TGF-β) superfamily acting as important multifactor players in the development of vertebrate skin and appendages. BMP signaling acts as a potent tumor suppressor in the skin, inhibiting mainly epidermal-derived and hair follicule-derived tumor formation [39]. Consequently, dysregulation of this pathway can lead to abnormal skin development and tumor formation [40]. A downregulation of three genes—*GPC3* (Glypican 3), *MSX2* (Msh homeobox 2), and *ILK* (Integrin-linked kinase)—involved in BMP signaling was observed in a whole-transcriptome microarray analysis of EBS-sev immortalized cells [22].

#### 2.1.6. T Helper Type 17 (Th17) Immune Response

T helper type 17 (Th17) cells play a role in adaptive immunity by protecting the body against pathogens by producing antimicrobial peptides, recruiting immunocytes via induction of chemokines, such as Chemokine (C-C motif) ligand 20 (CCL20), and repairing tissue by enhancing epithelial proliferation. The Th17 signaling pathway is tightly controlled and is terminated after infection is ablated and tissue repair is completed [41,42]. Higher levels of Th17 immune response markers such as IL-17, IL-21, and IL-22 were observed in the blister roof of patients with EBS-sev compared with controls by analysis of cytokine mRNA expression. Higher expression of TNFα, CCL20, IL-5, and IL-22 was also observed in the blister fluid. Moreover, blister roofs and fluid from patients with EBS-sev showed an increase in other cytokines inducing the differentiation of T cells toward Th17 cells, such as TGF-β, IL-6, and IL-21 [26]. Altogether, these results strongly suggest the involvement of the Th17 immune response in the EBS pathogenesis.

### 2.2. Keratins and Cell-Junction Components

Microarray experiments identified 129 differentially expressed probes/genes linked to cell–substrate or cell–cell junctions and/or other components of the cytoskeleton dysregulated in EBS keratinocytes. About 20 of these genes, encoding for cell junction components, including desmosomes, hemidesmosomes, adherent junctions, tight junctions, and gap junctions, are downregulated in the EBS-sev cell line compared with controls, with four of them also validated on the protein level [16]. Another study reported an upregulation of the two most abundant desmosomes components, desmoglein 3 (DSG3) and desmoplakin (DSP), in EBS-sev cells and interpreted this finding as a cellular response aiming at increasing the number and internal integrity of desmosomes [18]. Desmosomes are crucial for the integrity of the skin as they ensure mechanical strength by providing strong adhesion between cells. Several type 1 (K14, K16, K17) and type 2 (K5, K6a, K6b) keratins were upregulated in EBS-sev cells [18]. Another EBS expression microarray analysis on the epidermis of patients with EBS-sev identified several upregulated genes involved in epidermal keratinization, such as *SPRR4* and *KRT79* [20]. *KRT19* was significantly downregulated in one microarray analysis of EBS-sev keratinocytes versus controls indicating that KRT19 could constitute another useful biomarker in EBS [22]. 

The expression profile of EBS represents a lengthy list of dysregulated genes. Table 1 summarizes the results obtained from all studies cited above. 

## 3. Strategies to Improve and Alleviate EBS Manifestations

EBS remains one of the most well-understood inherited bullous diseases. However, it represents a challenge for treatment because of the dominant impact of keratin mutations and the broad number of mutations affecting either *KRT5* or *KRT14* (more than 200) [43]. The knowledge generated regarding the potential of inflammation components as therapeutic targets and the explosion of omics data combined with new genome editing approaches provided powerful insights for EBS. Added to symptom relief therapies, approaches targeting causative genetic defects are in development more than ever with the aim of curing or treating this disease. Table 2 regroups information regarding the therapeutic molecules tested in the context of EBS.

### 3.1. Therapeutic Molecules: Anti-Inflammatory

**Figure 2:**
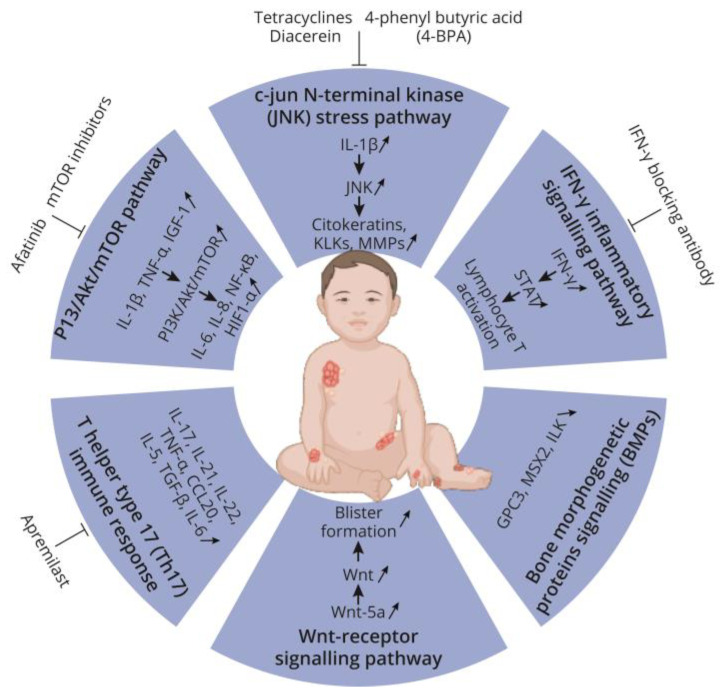
Inflammatory key pathways involved in blister formation in epidermolysis bullosa simplex (EBS) and their regulation by various therapeutic approaches.

The skin abnormalities in EBS are associated with inflammation and immune reaction via the production of chemokines and cytokines. This indicates possible therapeutic perspectives for decreasing chronic inflammation. Figure 2 offers an overview of key inflammatory pathways in EBS, identified in Section 2 of the present review, and the associated therapeutic approaches discussed below.

#### 3.1.1. Tetracycline Antibiotics

Tetracyclines are broad-spectrum bacteriostatic antibiotics used in dermatology for treating acne and are well known for inhibiting bacterial protein synthesis [44,45,46]. Tetracyclines are pluripotent drugs affecting various cellular functions, such as prion infectivity through direct interaction with proteinase-resistant forms of the prion protein [47]. One specific tetracycline, doxycycline, is an inhibitor of cell proliferation that modulates smooth muscle cell growth and matrix remodeling after arterial injury [48].

One clinical study suggests that oral treatment with tetracycline on patients with EBS-sev led to a significant reduction of blistering and less fragile epidermis [14,49]. Another trial on the K5−/− mouse model observed downregulation of matrix MMP13 and IL-1β expression and extended the survival of mice from less than one to up to eight hours when doxycycline was applied [14,50]. This shows the potential effect of doxycycline on transcription and its function as a potent MMP inhibitor, which could also be observed in a human model.

#### 3.1.2. Diacerein

Diacerein, is reported to block the release of active IL-1β by inhibiting plasma membrane-bound IL-1 converting enzyme (ICE) [51]. Treatment of keratinocytes from patients with EBS-sev with diacerein reduced the expression levels of K14 and IL-1β and the phosphorylation levels of JNK and, also, stabilized the intermediate IFs network upon heat shock [30]. One percent diacerein cream was tested for the topical treatment of EBS-sev in a pilot [52] and a phase II/III clinical trial and produced promising results with a reduction of more than 40% in blisters despite its transient effect [53]. A recent study reported no significant differences between the efficiency of diacerein 1% and placebo for patients with EBS, even when stratifying by severity [54]. These discordant results highlight the need for further investigations before stating on its efficiency as a reliable treatment for EBS clinical manifestations.

#### 3.1.3. Humanized IFN-γ Blocking Antibody

Therapeutic approaches aiming to block IFN-γ function might be a strategy for reducing skin fragility as it is usually increased in skin lesions. IFN-γ blocking antibodies were tested for the treatment of skin disorders, including dystrophic EB [55]. One mouse antibody targeting IFN-γ was successfully humanized without loss of affinity and applied to a cell model of EBS-sev. Results showed restoration of cell proliferation, increased cell–cell adhesion, accelerated wound closure in the presence of IFN-γ, and reduced IFN-γ mediated keratin aggregation [34,56]. This antibody represents a potentially valuable therapeutic agent for clinical trials as it is already humanized and can be directly administered in patients with EBS in effective doses, preventing side effects.

#### 3.1.4. mTOR Inhibitors

Using transcriptomic data for drug repositioning yielded novel treatment avenues for other skin conditions such as hidradenitis suppurativa [57], basal cell carcinoma [58], and dermatomyositis [59]. This strategy of selecting the best potential drugs whose effects on transcription would oppose the EBS signature led to the identification of the mTOR inhibitors as the best candidates. An independent pilot study of two patients with EBS treated with topical sirolimus for painful plantar keratoderma due to chronic blistering was conducted. Marked clinical improvement and a notable reduction of keratoderma were observed in EBS patients [24]. Sirolimus is known for its immunosuppressive properties. It forms a complex with FK binding proteins (FKBPs), ultimately binding with mTOR and inhibiting its function [60].

#### 3.1.5. 4-Phenyl Butyric Acid (4-PBA)

4-phenyl butyric acid (4-PBA) is a chemical chaperone facilitating protein folding which has been shown to reduce keratin aggregation in immortalized EBS-loc and EBS-sev primary keratinocytes after heat shock [61,62]. It was also reported that 4-PBA treatment led to a reduction of about 40% of the number of EBS-sev keratinocytes containing cytoplasmic keratin aggregates, highlighting the role of endogenous chaperone molecules in the degradation of mutated keratin molecules. Monitoring of ERK activation by western-blot analysis indicated that 4-PBA has a positive effect on ERK activation and reduces IL-1β expression [23,63].

#### 3.1.6. Apremilast (Anti-IL-17 Agent)

The presence of an immune infiltrate characterized by a Th17 response in the skin of EBS patients suggested that inhibitors of this pathway may be a promising new treatment [26]. Apremilast is a small molecule approved for the treatment of psoriasis, specifically inhibiting cyclic AMP phosphodiesterase-4 and Th1/Th17 activation [64,65]. Castela et al. 2019 tested the potential therapeutic benefits of apremilast in a small pilot study of three individuals with EBS-sev. They reported a rapid and sustained decrease in the number of blisters in all patients. Clinical trials should be the next step toward using this agent as a standard treatment for EBS patients.

#### 3.1.7. Afatinib (Epidermal Growth Factor Receptor (EGFR) Inhibitor)

Several pro-inflammatory cytokines, indicators of stress and wound-like activation of EBS keratinocytes, were found to be elevated in blister fluid from patients with EBS [66]. These include the epidermal growth factor (EGF) and its receptor EGFR, acting in a signaling pathway that is essential for keratinocytes migration and wound closure [67,68]. Treatment of EBS-sev cells in vitro with the FDA-approved EGFR inhibitor afatinib reduced keratin aggregates and favored stable filament networks. Afatinib treatment reverted the wound activation and healing migration state of EBS-sev disease model cells to a more quiescent state associated with improved keratin filament networks and cell–cell connections. This should improve the mechanical resilience of the epidermis and provide stability and resistance to mechanical stress [69].

### 3.2. Therapeutic Molecules: Beyond Anti-Inflammatory Mechanisms

Direct targeting of cellular structural abnormalities or lesions is another treatment strategy employed in EBS. Some of these molecules have the ability to directly influence post-translational mechanisms acting on pathways leading to impaired cellular integrity.

#### 3.2.1. Aluminium Chloride Hexahydrate

Aluminum chloride hexahydrate efficiency was first tested in two different case reports. These studies reported it as an effective treatment to prevent blisters in patients with EBS-loc [70,71]. A double-blind placebo-controlled randomized study on the administration of aluminum chloride hexahydrate in the same manner (20% solution in anhydrous ethyl alcohol) yielded no significant results regarding blister numbers in patients with EBS-loc [72]. The mechanisms underlying the possible success in two cases remain unclear and no further positive results were obtained for this molecule in EBS.

#### 3.2.2. Botulinum Toxins (BoNT)

Botulinum toxins (BoNT) are produced by the bacteria of the genra *Clostridium*. Their action inhibits the release of acetylcholine (ACh) and stops the signal for motion sent to muscle fibers [73]. BoNT, particularly type A and B (BTX-A and BTX-B), were used for treating the feet of patients with EBS-loc and EBS-sev in two different studies [74,75]. These studies showed improvement in blistering and alleviated pedal pain. One case report on a pediatric patient with EBS also showed pedal improvement for three parameters (blistering, pain, and odor) [76]. It is known that BoNT can have an effect on sensory nerves and, thus, affect the tenderness of the feet of patients with EBS, which seemingly helps diminish blister formation.

#### 3.2.3. Parthenolide (PN)

One plant derivate, parthenolide (PN), is known as a sesquiterpene lactone found in feverfew. PN possesses anti-inflammatory, redox-modulating, and epigenetic properties as well as selective toxicity for specific cancer cells [77]. A study by Sun et al. identified PN by high-throughput drug screening of molecules stabilizing IFs. They showed that PN normalized acetylation on keratins through augmented binding with NAD-dependent sirtuin 2 (SIRT2) [78]. This linked the acetylation pathways to the IF organization in a mice cellular model of EBS where disruption of keratin filaments is triggered by a mutation on *KRT14*. This resulted in increased cell adhesion and resistance to mechanical stress [78]. It is possible to assume that some clinical manifestations of EBS, such as blistering, could be alleviated with PN. Clinical trials are still needed before the effect on the cells and skin of EBS patients is proven.

#### 3.2.4. PKC412 Kinase Inhibition

This kinase is also known as midostaurin, a targeted therapy used in combination with chemotherapy for the treatment of acute myeloid leukemia [79]. This molecule has also been identified as normalizing the level of disruption of the keratin filament caused by a mutation in *KRT18* in mice [80]. Following this rationale, Rietscher et al. used this molecule to reduce keratin aggregates in keratinocytes derived from patients with a causal mutation on *KRT14*. Results showed a lowering of 40% in keratin aggregates as well as lesser phosphorylation on keratin, both leading to strengthened cellular cohesion [81]. This kinase directly affects the phosphorylation level of keratins because of the high accessibility of the non-α-helical domains in the head and tail of keratins [81,82]. This lowered phosphorylation, in turn, allows for a better organization of intracellular keratin filaments and indirectly affects DSP, a protein useful in cell–cell contact sites [81]. This modification of a post-translational mechanism holds promising outcomes for EBS treatment.

**Table 2:** Molecules tested in epidermolysis bullosa simplex (EBS).

Molecule	Reference	Type of Study	Patients	Vehicle	Target	Outcomes	Perspectives
**4-PBA**	[62]	In vitro	EBS-loc, EBS-sev	1 mM solution	Immortalized keratinocytes	Reduction of keratin aggregates upon heat shock	Reversing protein aggregates and reducing tissue fragility
	[63]	In vitro	EBS-sev	1 mM solution	Immortalized keratinocytes	Reduction of keratin aggregates and amelioration of inflammatory phenotype	Assay with low doses to avoid toxicity
	[23]	In vitro	EBS-sev	1 μM solution	Keratinocytes	Reduction of around 40% of keratin aggregates	Reversing protein aggregates and reducing tissue fragility
**Afatinib**	[69]	In vitro	EBS-sev	1 μM solution	Immortalized keratinocytes	Reduction of keratin aggregates, induction of quiescent state to cells	Optimize afatinib for therapy (reduce adverse side effects when used in cancer therapy)
**Aluminium chloride hexahydrate**	[70]	Case report	EBS-loc	20% in alcohol	Feet	Prevent new blisters	Study other cases to establish a pattern of efficiency
	[71]	Case report	EBS-loc	20% in alcohol	Hands and feet	Prevent new blisters	Study other cases to establish a pattern of efficiency
	[72]	Therapeutic assay	EBS-loc	20% in alcohol	Feet	No significant reduction of blisters	Study the effect on more severe subtypes
**Apremilast**	[26]	Therapeutic assay	EBS-sev	Oral treatment (10 mg/day to 30 mg twice/day)	Skin	Rapid and sustained (7–10 months) improvement in skin lesions	Trial with a higher number of patients (placebo–control)
**Botulinum toxins**	[74]	Case report	EBS-loc	100 U BTX-A (Botox) in saline	Feet	65% reduction in blister surface area, decrease in pain and perspiration	Trial with a higher number of patients (placebo–control), testing higher concentration of the molecule
	[75]	Therapeutic assay	EBS-loc, EBS-sev	170–700 U BTX-A (Dysport) in saline, 2500 U BTX-B (Neurobloc) in saline	Feet	Improvement in blistering and pedal pain	Trial with a higher number of patients (placebo–control)
	[76]	Case report	EBS	100 U BTX-A (Botox) in saline	Feet	Reduction of blisters, smaller blisters, decreased pedal pain and odor	Treatment for pain management and improved quality of life
**Diacerein**	[30]	In vitro	EBS-sev	10 μg mL^−1^ solution	Keratinocytes	Stabilization of the IF network and reduction of inflammatory components	Trial with EBS-sev patients
	[52]	Therapeutic assay	EBS-sev	1% cream	Armpits	Reduction of blisters	Trial with a higher number of patients
	[53]	Phase 2/3 clinical trial	EBS-sev	1% cream	Skin	More than 40% reduction in blister number	Trial with a higher number of patients and more invasive data acquisition
	[54]	Therapeutic assay	EBS-sev, EBS-intermed	1% cream	Skin	No significant improvement compared with vehicule cream alone	Focus on EBS-sev individuals with a bigger sample size
**Doxycycline**	[14]	In vivo	K5−/− mice	50 μg/mL solution with 5% sucrose	Skin	Downregulation of MMPP13 and IL-1β	Screening similar compounds for additional targets
**INF-γ blocking antibodies**	[34]	In vitro	Cell model of EBS-sev	Humanized monoclonal antibody	Keratinocytes	Reversing effect of IFN-γ (less keratin aggregates, restored cell proliferation, increased cell–cell adhesion, accelerated wound closure)	Promising therapy for patients with EBS or other skin disease (in vivo assays)
**mTOR inhibitors**	[24]	Therapeutic assay	EBS	2% sirolimus ointment	Feet	Reduction of blisters and keratoderma	Drug repositioning based on transcriptomic signature in EBS
**Parthenolide**	[78]	In vitro	Mice cell model of EBS	5 µM solution	Keratinocytes	Increasing cell adhesion and resistance to mechanical stress	Trials with keratinocytes of patients to assess efficiency
**PKC412 kinase inhibitor**	[81]	In vitro	EBS-sev	1 µM solution	Immortalized keratinocytes	Reduction of 40% of keratin aggregates and strengthening of cellular cohesion	Pretesting on animal models and skin explants before oral or topic administration in EBS patients
**Tetracycline**	[49]	Therapeutic assay	EBS-sev	Oral treatment (1500 mg/day)	Skin	Reduction of blisters and less fragile epidermis (dose-dependant response)	Trial with a higher number of patients

Abbreviations—BTX-A: botulinum toxin type-A; BTX-B: botulinum toxin type-B; EBS-intermed: intermediate EBS; EBS-loc: localized EBS; EBS-sev: severe EBS; IL-1β: interleukin-1 β; MMPP13: matrix metallopeptidase 1.

### 3.3. Genome Editing Approaches

#### 3.3.1. RNA Trans-Splicing

Studies using spliceosome-mediated mRNA trans-splicing (SMaRT) have proved its feasibility to correct mutations at the mRNA level by replacing up to 4.5 kb of a sequence [83]. The basis of SMaRT is the engineering of an RNA trans-splicing molecule (RTM) containing the coding region to be replaced. This method was used to correct a mutation in exon 1 of *KRT14* by using a previously reported 5′-trans-splicing module replacing exons 1–7 of the *KRT14* mRNA [84,85], Accordingly, the RNA trans-splicing module was transduced into an EBS-sev patient-derived keratinocyte cell line. Subsequently, skin equivalents were generated with these corrected cells and grafted onto the backs of mice. These skin equivalents revealed a stable, well-differentiated epidermis, thus showing the potential of trans-splicing as a therapeutic approach for EBS. The major advantage of trans-splicing is that one RNA trans-splicing module can be employed to correct multiple variants in several exons, eliminating the need to design specific RTM for each mutation.

#### 3.3.2. Transcription Activator-like Effector Nucleases (TALENS)

Programmable nucleases, named transcription activator-like effector nucleases (TALENs), are an approach consisting of the induction of DNA double-strand breaks (DSBs). This activates the endogenous repair machinery, resulting in gene disruption via nonhomologous end joining (NHEJ). Aushev et al. showed that TALENs efficiently disrupted the *KRT5* locus in immortalized EBS-intermed and EBS-sev human epidermal keratinocytes, with 25% and 29% of clones showing NHEJ, respectively [66]. Inactivation of the mutant *KRT5* allele resulted in the elimination of structural abnormalities in IFs and the reduction of cytoplasmic keratin aggregates upon stress.

#### 3.3.3. CRISPR-Cas9

CRISPR/Cas9 gene editing allows for the introduction of precise changes to specific sequences of genes. It has become a widely used tool in medical research for the correction of disease-associated mutations [86,87,88]. Kocher et al. corrected a causal hotspot mutation in exon 6 of *KRT14* resulting in EBS-sev phenotype in vitro [89]. A double-nicking strategy targeting intron 7 was used, followed by homology-directed repair (HDR). Co-delivery into keratinocytes of a Cas9 D10A nickase (Cas9n), a predicted single guide RNA pair specific for intron 7, and a minicircle donor vector harboring the homology donor template resulted in a recombination efficiency of more than 30% and correction of the mutant *KRT14* allele without off-target activity.

Bchetnia et al. succeeded in eliminating the mutant protein K5 by disrupting the mutant allele while leaving the wild-type functional [90]. The mutant allele was disrupted for one heterozygous EBS-sev pathogenic variation within *KRT5* by introducing a DNA cleavage in the patient’s keratinocytes using a stringent CRISPR-Cas9 system. Results showed successful stringent mutant allele-specific silencing at the DNA and RNA level, indicating permanent mutant allele-specific inactivation. Edited EBS-sev keratinocytes produced a lower amount of K5 and K14 proteins compared with non-edited EBS cells with no disturbance of cellular properties observed.

## 4. Why Therapeutic Approaches Are Difficult to Find in Epidermolysis Bullosa?

Genetic variations in EBS are caused by several mutations, leading to subtypes with variable severity [12,13]. This genetic variability is challenging for the development of universal therapeutic approaches. Another important challenge is that specific molecular targets across all subtypes of EBS remain elusive due to the diversity of genetic mutations involved (as well as the difficulty of identifying a readout). In addition, the constant presence of wounds in EBS patients can trigger immune responses and inflammation, complicating treatment efforts. Anti-inflammatory approaches might exacerbate these issues, and finding ways to modulate the immune response without causing further damage is complex. Finally, as for other rare disorders, the scarcity of EBS patients means that clinical trials for the development and validation of potential therapies are difficult to establish [91]. The results obtained from existing trials are encouraging but difficult to interpret for all the phenotypes observed. Despite these challenges, new technologies in gene editing, regenerative medicine, high-throughput molecule screening, drug repurposing, and innovative wound care strategies are promising potential therapeutic avenues.

## 5. Conclusions

This review summarized the differentially expressed mRNAs in EBS and predicted the biological processes and signaling pathways involved in this skin disorder. The cellular pathology of most EBS subtypes is associated with the fragility of the IFs network, cytolysis of the basal layer of the epidermis, or attenuation of hemidesmosomal/desmosomal components. Despite the limited overlap of genes identified, major evidence in the literature supports the implication of inflammation in EBS. Such review will undoubtedly have a bearing on the development of new therapeutic strategies and accelerate the translation of these approaches to the clinic. Although no single therapy has achieved complete success, it seems probable that combining different therapeutic principles will yield the best outcomes and improve the quality of life of patients with EBS.

## Figures and Tables

**Table 1:** Epidermolysis bullosa simplex (EBS) gene expression profiling studies under normal growth conditions.

Reference	Model/Cell Lines	Upregulated Genes		Downregulated Genes	
[14]	WT mouse skin vs. K5^-^−/− mouse skin	Gene symbol	Method	Gene symbol	Method
		***CXCL1***, *CXCL2*, *HPRT*, ***KRT6a***	MA		
		*IL6*, ***IL1-β***	MA, RTq-PCR		
[16]	WT vs. EBS-sev KEB-7	*ACTN1*, *AJAP1*, *CDH2*, *CDKN2A*, *CST6*, *DBN1*, *FLNB*, *FN1*, *GNA11*, *IGSF4*, *KIAA0992*, *KRT18*, *KRT8*, *KRTHB1*, *LMNB2*, *MYL9*, *PODXL*, *PRSS2*, *RHOA*, *TGM2*, *TNC*, *TPM2*, *TUBB2*, *VIM*	MA	*ABLIM1*, *AMOTL2*, *ANXA1*, *ARHGDIB*, *CLDN4*, *COL17A1*, *CSTA*, *CTGF*, *CXADR*, *DDR1*, *DST*, *EGFR*, *ENC1*, *FRMD4B*, *GABARAPL1*, *GPNMB*, *HSPB1*, *IL18*, ***IL8***, *KRT13*, ***KRT15***, ***KRT16***, ***KRT19***, ***KRT5***, *LPXN*, *LY6D*, *MTSS1*, *OLR1*, *PCDH7*, *PDLIM1*, *PDZK3*, *PFN2*, *RND3*, *SAA1*, *SCEL*, *SLC9A3R1*, *SNCG*, *PRR1B*, *STEAP1*, *TSPAN1*	MA
				*CDH1*, *GJB5*, *ITGA6*, *LAMA5*, *LAMB3*, *PPL*	MA, RTq-PCR
				*CLDN3*, *CLDN7*, ***DSC2***, *PKP1*	RTq-PCR
				***DSG3***, *GJA1*, *JUP*	MA, RTq-PCR, WB, IF
				***DSP***	MA, WB, IF
	WT vs. EBS-sev KEB-1			*GJA1*, ***DSG3***, ***DSP***, *JUP*	WB
	WT vs. EBS-loc KEB-4	*ACTA2*, *AJAP1*, *ANK3*, *CLDN3*, *CLDN7*, *GSN*, ***KRT14***, ***KRT15***, ***KRT16*****, *****KRT17***, *KRT4*, *LGALS7*, *HLA-B*, *HLA-C*, *NEFH*	MA	*PCDH7*, *BASP1*, *CTGF*, *CYR61*, *DST*, *ENC1*, *FRMD4B*, *IL18*, ***KRT19***, *LPXN*, *OLR1*, *RDX*, *RND3*, *STK6*	MA
[17]	WT mouse skin vs. K5−/− mouse skin	*MCP-1*, *CCL2*, *MIP-3a/CCL20*, *MIP-3b/CCL19*, *CXCL16*			
[18]	WT vs. EBS-sev KEB-7	*ACSL3*, *ANXA2*, *ARPC3*, *CAV1*, *CCT5*, *EEF1G*, *EIF1*, *ENO1*, *GLUD1*, *H2AFZ*, *HNRPDL*, *SP90AB1*, *MAN2A1*, *PDCD4*, *RAB27B*, *RPL13*, *RPL19*, *RPL23A*, *RPL28*, *RPL31*, *RPL4*, *RPL5*, *RPL7A*, *RPLP0*, *RPS18*, *RPS2*, *PS25*, *RPS3*, *RPS4X*, *RPS6*, *RPS8*, *SFN*, *TAX1BP1*, *TMBIM6*, *TPI1*, *TPT1*, *EEF1A1*, *GSN*, *HNRNPA1*, ***KRT5***, ***KRT6A***	SSH		
		*ANXA8*, *DDIT4*, ***DSG3***, ***DSP***, *F3*, ***KRT14***, ***KRT16***, ***KRT17***, ***KRT6B***, *MALAT1*, *PERP*, *TXNIP*, *UBE2K*, *YWHAZ*	SSH, RTq-PCR		
[20]	WT human epidermis tissue vs. EBS-sev and EBS-loc human epidermis tissue	*SPRR2B*, *AREG*, *BDP1*, *NR4A2*, *GAL*, *LYVE1*, *PSG4*, *H19*, *PDE6A*, *C5orf27*, *THRSP*, *FAR2*, *AADACL3*, *CRAT*, *AGR2*, *IGFL4*, ***KLK6***, *PM20D1*, *ATP12A*, *FSIP2*	MA		
		*AWAT2*, *DGAT2L6*, *FADS2*, *ACSBG1*, *SPRR4*, *KRT79*	MA, RTq-PCR		
		*ELOVL3*	MA, RTq-PCR, WB		
	WT human epidermis vs. EBS-sev human epidermis	*FADS1*, *CYP4F8*, *AWAT1*, *ALOX15B*, *ACSM3*, *SOAT1*, *SLC27A2*, *HAO2*, *INSIG1*, *KRTAP5-8*, *KRT25*, *KRT71*, *KRT74*, *KRT27*, *TCHH*, *CHI3L1*, *MUC1*, *SLCO4C1*, *TGIF2LX*, *IGLJ3*, *IGJ*, *IGHA1*, *TMEM56*, *LRCC37A2*, *THRSP*, *FAR2*, *AADACL3*, *CRAT*, *AGR2*, *IGFL4*, ***KLK6***, *PM20D1*, *ATP12A*, *FSIP2*			
		*AWAT2*, *DGAT2L6*, *FADS2*, *ACSBG1*, *SPRR4*, *KRT79*	MA, RTq-PCR		
		*ELOVL3*	MA, RTq-PCR, WB		
[30]	WT immortalized keratinocytes vs. immortalized EBS-sev cell lines (KEB-7, EBDM-1)	***KRT14***,***IL-1b***, ***KRT6A***	SqRT-PCR		
[21]	WT immortalized keratinocytes vs. immortalized EBS-sev cell lines (KEB-7, EBDM-1)	***KLK6***, *KLK8*, *KLK10*, *KLK11*, *KLK13*, *KLK14*, *MMP1*, *MMP13*, *WIPF1*, *ARHGEF4*, *ARHGEF37*, *CDC42BPG*, ***KRT6B***	MA		
		***KLK5***, *KLK7*, ***KRT14***, ***KRT15***, ***KRT16 KRT17***, ***KRT5***	MA, sqRT-PCR, WB		
		*MMP7*, *MMP9*, *MMP19*, *ARHGEF9*, *DSC1*, ***DSC2***, *DSC3*, *DSG1*, ***DSG3***, *DSG4*, *GJA1*, *GJB2*, *GJB6*, ***CXCL1***, *CXCL8/**IL-8***, *CXCL14*	MA, sqRT-PCR		
[22]	WT vs. EBS-sev KEB-7	*KGFLP1*, *ANKRD2*, *PIK3R3*, *PTPN20A*, *FAM21A*, *APBB2*, *DUOX1*, *ZNF627*, *MCOLN2*, *APOB*, *AJAP1*, *BNC2*, *LOC100652860*, *FKTN*, *TMEM204*, *BMS1P1*, *MAN1A1*, *STOX1*, *RPL10*, *CDC144C*, *NOX5EVC2*, *PTPN20C*, *PTPN20B*, *NID1*, *ASAH2*, *FLJ20444*, *TP53INP1*, *FRG1B*, *MSLN*, *DENND1B*, *IFFO2*, *STRBP*, *DSEL*, *AOX1*, *LRP12*, *ADHFE1*, *FAM21D*, *SNORD64*, *FAM21A*, *EFEMP1*, *TSPYL5*, *RNF212*, *DDX43*, *ZNF136*, *CCDC144A*, *CCDC144A*, *ZNF700*, *BGN*, *H2AFY2*, *SNORD116-21*, *HOXA9*, *RPS23*	MA	*MIR492*, *H19*, *EYA4*, *TMPRSS15*, *ITGBL1*, *EDIL3*, *CDR1*, *NEFL*, *SMOC2*, *GHR*, *TFPI2*, *ARHGAP28*, *NNMT*, *SOX2*, *HIST1H*, *PCCA*, *ZNF570*, *CDK14*, *MEST*, *CYP7B1*, *GALNTL4*, *CRIP2*, *IPO7*, *SAAL1*, *KRTCAP3*, *FAM159A*, *EYA1*, *CDC25B*, *NKX2-6*, *HTATIP2*, *ILK*, *ACSF2*, *PDZD2*, *CENPH*, *TOX*, *VSTM2L*, *SYT17*, *SLC7A2*, *IKZF3*	MA
		*TDRD12*, *NEFH*, *NLRP2*, ***KLK5***, *ENPP1*, *ZFP42*, *DKK1*, *CYYR1*, *C10orf99*, *SYCP2*, *PRICKLE1*, *SLC44A5*, *PLA2G7*, *MOXD1*, *WNT5A*, *WISP3*, *ARHGEF9*, *HSD17B11*, *ADAMTSL3*, *FAM102B SGMS1*, *ARHGAP29*, *SLC15A2*, *ROBO1*, *ERCC6*, *NREP*, *KIAA1324L*, *ROR1*, *ZFAND4*, *SELENBP1*, *NF334* *PTPN20C*, *IRX4*, *PTPN20A*, *PNMAL1*, *NID1*, *ZNF32*, *AHI1*, *ELAVL2*, *SLC16A9*, *FAM25A*, *MAPK8*, *CDC14B*, *GABPB2 TCHH*, *LMF1*, *CSTF2T*, *SGK1*, *UAP1*, *POPDC2*, *ZNF502*	MA, RTq-PCR	***KRT19***, *KYNU*, *PDZK1*, *OLFM4*, *SLC38A4*, *BST2*, *PPARGC1A*, *GALNT5*, *FKBP10*, *GIPC2*, *AMOT*, *ZNF114*, *CLEC2B*, *FAM198B*, *SLC2A3*, *CAPNS2*, *TBX18*, *LRCH2*, *NEFM*, *CPT1C*, *ZNF43*, *LY75*, *GLDC*, *TMTC1*, *SLCO1B3*, *SLC6A14*, *SLC24A3*, *EPSTI1*, *SATB2*, *HSD17B2*, *AKR1B10*, *GPC3*, *IFITM3*, *HOXD10*, *MSX2*, *IL17RB*, *BLMH*, *SLC9A2*, *CPNE1*, *WDR17*, *RB1*, *DPYD*, *PRTFDC1*, *GLRX*, *PPP1R16B*, *GTF2H2D*, *REPS2*, *GPR143*, *GTF2H2*, *CYP7B1*, *BCL11A*, *MERTK*, *PRDM5*, *ACOXL*, *AHCY*, *ARMCX2*, *PAX6*, *HOXD11*, *SMARCA1*, *IFI44L*, *PITRM1*, *NAP1L5*, *PIGU*	MA, RTq-PCR
[23]	WT human keratinocytes vs. EBS-loc and EBS-intermed keratinocytes	*ACOT1*, *ALDH1A3*, *ALOX15B*, *ALPL 6*, *ANGPTL4*, *ASPN 34*, *ATP6V0A4*, *BEX1*, *C6orf223*, *CARD17*, *CCDC9B*, *CCL2*, *CCL20*, *CD99L2*, *CNIH3*, *CNTN3*, *COL1A1*, *COL6A6*, *CRYBB2P1*, *CXCL10*, *CXCL11*, *CXCL5*, *CXCL6*, *CYP1A1*, *DAAM2*, *DLK1*, *ECHDC1*, *ELN*, *FIBIN*, *FMOD*, *FNDC1*, *G0S2*, *GALNT14*, *GALNT16*, *HLA-DRB1*, *HMGB3*, *HS3ST2*, *HSD17B2*, *IER3*, *IFI27*, *IFI44*, *IL1A*, *IL1B*, *IL7R*, *INHBA*, *INSYN2B*, *ITGA10*, *ITGBL1*, *KRT6B*, *LAMB3*, *LIF*, *LINC00520*, *LINC-PINT*, *LOC100996732*, *LOX14*, *LSP1P4*, *LSP1P5*, *MANCR*, *MFAP4*, *MT1L*, *MX2*, *NFIX*, *NOV*, *NPAS2*, *NPIPB12*, *NPIPB13*, *NPIPB3*, *NPIPB5*, *NUPR1*, *LFML2B*, *PCDHA6*, *PDGFRB*, *PLD5*, *POSTN*, *PTGS1*, *RTEL1-TNFRSF6B*, *SAA1*, *SAA2*, *SAA2-SAA4*, *SERPINB2*, *SFRP2*, *SLC9A7*, *SMOC1*, *SMOC2*, *ST6GAL2*, *SYNDIG1*, *TAC1*, *TAGLN*, *TFCP2L1*, *TMEM255B*, *TNFRSF6B*, *TNMD*, *TPST1*, *TRNP1*, *VNN1*, *WIPI1*, *XDH*			RNA seq

Abbreviations—EBDM-1: EBS severe disease phenotype cell line with K14 R125H mutation; EBS-loc: localized EBS; EBS-sev: severe EBS; IF: immunofluorescence; K5: keratin 5; KEB-1: cell line with K5 E475G mutation; KEB-4: cell line with K14 V270M mutation; KEB-7: cell line with K14 R125P mutation; MA: microarray; RTq-PCR: real-time quantitative PCR; sqRT-PCR: semi-quantitative real-time PCR; SSH: suppression subtractive hybridization; WB: Western blot; WT: wild-type. In bold—genes found to be regulated in multiple studies.

## Data Availability

No new data were created or analyzed in this study. Data sharing is not applicable to this article.
